# Impaired SIRT1 activity leads to diminution in glomerular endowment without accelerating age‐associated GFR decline

**DOI:** 10.14814/phy2.14044

**Published:** 2019-05-13

**Authors:** Ashley R. Bellin, Yanling Zhang, Kerri Thai, Norman D. Rosenblum, Luise A. Cullen‐McEwen, John F. Bertram, Richard E. Gilbert

**Affiliations:** ^1^ Keenan Research Centre Li Ka Shing Knowledge Institute St. Michael's Hospital Toronto Ontario Canada; ^2^ Development and Stem Cells Program Monash Biomedicine Discovery Institute, and Department of Anatomy and Developmental Biology Monash University Clayton Victoria Australia; ^3^ Hospital for Sick Children Research Institute Toronto Ontario Canada

**Keywords:** Aging, glomerular endowment, glomerular filtration rate, SIRT1

## Abstract

Glomerular filtration rate (GFR) declines with age such that the prevalence of chronic kidney disease is much higher in the elderly. SIRT1 is the leading member of the sirtuin family of NAD ^+^‐dependent lysine deacetylases that mediate the health span extending properties of caloric restriction. Since reduction in energy intake has also been shown to decrease age‐related kidney disease in rodents, we hypothesized that a diminution in SIRT1 activity would accelerate the GFR decline and structural injury with age. To test this hypothesis, we compared changes in the kidney structure and function in control mice and mice that carry a point mutation at a conserved histidine (H355Y) of SIRT1 that renders the enzyme catalytically inactive. Taking advantage of this mouse model along with the disector/fractionator technique for glomerular counting and direct measurements of GFR by inulin clearance, we assessed the impact of SIRT1 inactivity on kidney aging. At 14 months of age, SIRT1 catalytically inactive (*Sirt1* ^*Y/Y*^) mice had lower GFRs and fewer glomeruli than their wild‐type *(Sirt1* ^*+/+*^) counterparts. This was not, however, due to either accelerated GFR decline or increased glomerulosclerosis and loss, but rather to reduced glomerular endowment in *Sirt1* ^*Y/Y*^ mice. Moreover, the compensatory glomerular hypertrophy and elevated single nephron GFR that customarily accompany reduction in nephron number were absent in *Sirt1* ^*Y/Y*^ mice. These findings suggest a role for SIRT1 not only in determining nephron endowment but also in orchestrating the response to it.

## Introduction

Decline in organ function, an almost inevitable consequence of aging, contributes to much of the morbidity and mortality in the industrialized world. In the kidney, this is manifested by a progressive reduction in the number of fully functioning glomeruli as a consequence of sclerosis which can lead to atrophy and reabsorption (Denic et al. 2017a). Accompanying these structural changes, kidney function falls with age with an annual decline in glomerular filtration rate (GFR) of around 1.0 mL/min/1.73 m^2^/year (Choudhury and Levi 2016). As such, aging alone or in conjunction with age‐related disorders, such as diabetes and hypertension, contributes to the high prevalence of chronic kidney disease (CKD) in the general population. In the United States, for instance, while 11% of the adult population have an eGFR < 60 mL/min/1.73 m^2^/year (CKD3+) the prevalence is age‐dependent such that this degree of renal impairment is seen in 8.5% of those aged 20–39 years, 12.6% of those aged 40–59 years, and 39.4% of those aged >60 years (Coresh et al. 2003). These statistics notably have implications not only for the development of kidney failure but also for cardiovascular disease where the likelihood of death increases exponentially with loss of GFR (Fox et al. 2012).

Calorie restriction is one of the very few interventions that has been shown to extend life span and organ health in a wide range of organisms from yeast to fruit flies through to mice. Pivotal studies, conducted almost 20 years ago, showed that this process is mediated by silent information regulator‐2 (*Sir2*) in lower organisms (Lin et al. 2000) and its mammalian orthologue, *Sirt1* in mammals (Cohen et al. 2004). These nicotine adenine dinucleotide (NAD^+^)‐dependent lysine deacetylases are ubiquitously expressed enzymes that modulate a myriad of cell functions that center on adaptation to environmental stressors by regulating intermediary metabolism, mitochondrial function, circadian rhythmicity, and DNA repair (Guarente and Franklin 2011).

Since reduction in energy intake has been shown to not only extend life span but to also decrease age‐related kidney disease in rodents (Jiang et al. 2005), we sought to study the effects of SIRT1 on kidney aging, taking advantage of a well‐established mouse strain that carries a point mutation at a conserved histidine (H355Y) of *Sirt1* and renders it inactive (Boily et al. 2008; Caron et al. 2014).

## Materials and Methods

### Animals

Three genotypically different mouse strains were studied. Mice carrying a point mutation (H355Y) that ablates the catalytic activity of Sirt1, as previously described (Boily et al. 2008; Seifert et al. 2012), on an outbred (129xCD1/KJ325) background (generous gift of Dr. M. McBurney, Ottawa), served as a model of Sirt1 inactivity. As reported previously (Seifert et al. 2012), mice carrying the mutant sirt1^Y^ allele on the 129/SvJ background were outcrossed to CD‐1 mice, and heterozygous mice were intercrossed with viable offspring genotyped at 3 weeks of age. Mice homozygous for the mutant allele, sirt1^Y/Y^ were fewer than the expected 25% of the offspring from these crosses. Wild‐type sirt1^+/+^ and heterozygous sirt1^+/Y^ mice were present in the expected 1:2 ratio.

Homozygous *Sirt1* ^*Y/Y*^ mice with two nonfunctional Sirt1 alleles (Y) were then compared with heterozygous *Sirt1* ^*+/Y*^ and wild‐type *Sirt1* ^*+/+*^ mice. Because of their propensity to develop more advanced disease, only male mice were studied (Neugarten and Golestaneh 2013).

As a consequence of the median survival time of 60 weeks among SIRT1‐deficient mice fed an ad libitum diet (Mercken et al. 2014), mice were not aged beyond 14 months.

In Study 1 of aged mice, 25 animals (9 *Sirt1* ^*+/+*^, 8 *Sirt1* ^*+/Y*^, and 8 *Sirt1* ^*Y/Y*^) were aged to 14 months with a subset of 12 (5 *Sirt1* ^*+/+*^, 3 *Sirt1* ^*+/Y*^, and 4 *Sirt1* ^*Y/Y*^) randomly selected for longitudinal GFR measurements at 3, 6, 9, and 12 months of age. Another subset of 12 animals (4 *Sirt1* ^*+/+*^, 4 *Sirt1* ^*+/Y*^, 4 *Sirt1* ^*Y/Y*^) from the original 25 mice were randomly selected to assess their level of activity and body temperature with an implantable transmitter (HD‐X11, Data Sciences International, St. Paul, MN), as previously described (Zhang et al. 2017). All 25 mice were perfused with formalin at termination to preserve kidney structure for the counting of glomerular number and the assessment of glomerular volume. The kidneys of two animals were inadequately stained and therefore excluded from further analyses.

In Study 2, 27 male mice (9 *Sirt1* ^*+/+*^, 9 *Sirt1* ^*+/Y*^, and 9 *Sirt1* ^*Y/Y*^) aged 4 weeks were similarly perfused with formalin for glomerular counting and glomerular volume assessments with 22 of them randomly assigned to undergo GFR measurement (7 *Sirt1* ^*+/+*^, 7 *Sirt1* ^*+/Y*^, 8 *Sirt1* ^*Y/Y*^) prior to sacrifice.

Breeding and maintenance of this mutant strain were conducted at St. Michael's Hospital Research Vivarium with all procedures approved by the St. Michael's Hospital Animal Ethics Committee in accordance with the Guide for the Care and Use of Laboratory Animals (Council 2011). Mice were kept on a 12‐h light/dark cycle with free access to food and water. All procedures were conducted during the light phase cycles. At the end of the study, the mice inhaled 2–3% isoflurane before cervical dislocation.

### SIRT1 activity

SIRT1 catalytic activity was assessed by quantifying the acetylation of one of its substrates, H3K9/14, as previously reported (Bugyei‐Twum et al. 2018). In brief, total protein was extracted with ice‐cold radioimmunoprecipitation buffer containing a protease inhibitor mixture and quantified with a Bio‐Rad Protein Assay Reagent. Protein samples were then separated by SDS‐PAGE and transferred onto nitrocellulose membranes. Membranes were blocked with 5% skim milk in TBS‐T and probed with the acetyl‐histone H3 (Lys9/Lys14) antibody (Cell Signaling Technologies #9677, Danvers, MA). Anti‐tubulin antibody conjugated to horseradish peroxidase (Cell Signaling Technologies) was used as a loading control so that abundance of acetyl‐histone H3 (Lys9/Lys14) was quantified by enhanced chemiluminescence detection system (Amersham Biosciences, Amersham, UK) relative to tubulin.

### Kidney function

Glomerular filtration rate was assessed by fluorescein isothiocyanate (FITC)‐inulin clearance, as previously described (Qi et al. 2004). Briefly, the mice were injected in the tail vein with 3.74 *μ*L/g body weight FITC‐inulin. Saphenous vein blood was sampled at predetermined time points following injection. Plasma was buffered to pH 7.4 using HEPES solution and the concentration of FITC‐inulin was determined using a fluorescence assay with a Spectramax M5e microplate reader (Molecular Devices, Sunnyvale, CA) with excitation and emission wavelength settings of 485 nm and 538 nm, respectively. GFR was then calculated using a two‐phase exponential decay curve and nonlinear regression method in which GFR = *I*/(*A*/*α + B*/*β*), where *I* is the amount of FITC‐inulin injected, A and B are *y*‐intercepts for the two decay rates, and *α* and *β* are decay constants for the distribution and elimination phases.

Estimated single nephron GFR (eSNGFR) was calculated by dividing GFR by twice the number of glomeruli enumerated in a single kidney (eSNGFR = GFR/(glomerular number × 2).

Albuminuria was assessed using a Mouse Albumin ELISA Assay kit with 24‐h urine specimens obtained from mice housed individually in metabolic cages just prior to termination.

### Tissue preparation and histochemistry

Following termination by cervical dislocation, mice were perfused with a solution of PBS and heparin, followed by 10% neutral buffered formalin. The left kidney was removed and immersion fixed in 10% neutral buffered formalin for a minimum of 48 h before being processed and embedded in paraffin. The kidney was later sectioned and stained histochemically with either *A. hypogaea* peanut agglutinin (PNA) or periodic acid Schiff (PAS), to assess renal structure as described below.

### Glomerular number

**Figure 1:**
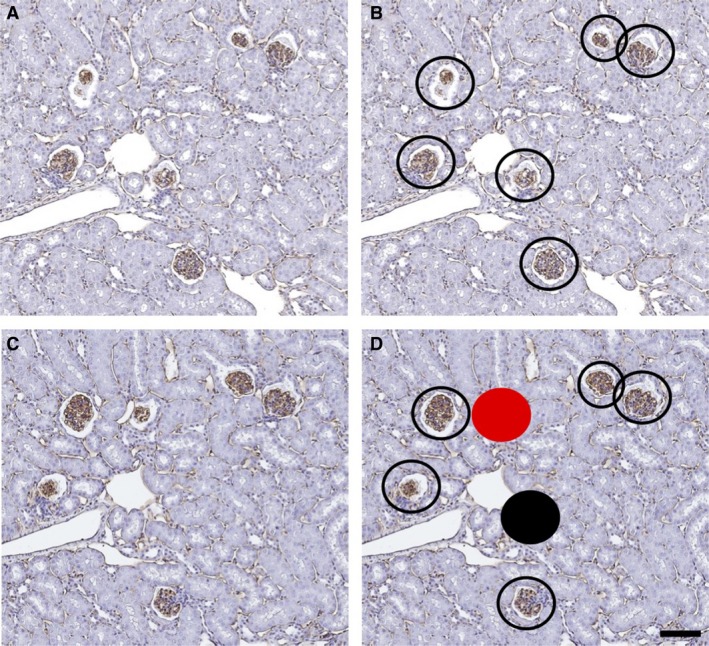
Representative histological images used for estimating the number of glomeruli per kidney (Nglom). (A) Image of PNA‐stained 14‐month mouse section (*n*th), identifying the presence of PNA‐positive podocytes. (B) All glomeruli with PNA‐positive podocytes are marked with open circles. (C) Image of PNA‐positive *n*th* *+* *2 section. (D) Image of *n*th* *+* *2 section with overlay of glomeruli identified on the *n*th section. Glomeruli present in the *n*th section not present in the *n*th* *+* *2 section are indicated (filled black circles). Glomeruli not present in the *n*th section but present in the *n*th* *+* *2 section are also indicated (filled red circles). Both sets of filled circles (red and black) are counted according to the disector principle (*Q* ^−^). Scale bar = 100 *μ*m.

Glomerular number was calculated using the current gold standard, physical disector/fractionator stereological method for deriving glomerular number in developing and adult kidneys, as previously published (Cullen‐McEwen et al. 2011). Briefly, the kidneys were exhaustively sectioned at a nominal thickness of 5 *μ*m, with two section pairs collected every 40–60 sections depending on the kidney size. A sampling fraction was calculated to achieve approximately 10–12 section pairs consisting of *n* (reference section) and *n *+* *2 (lookup section). Selected sections were stained with PNA in order to distinguish glomeruli. Slides were then scanned at 20× using an Aperio AT2 Scanner (Leica Biosystems Inc.) and counted using a computer‐assisted image analysis program, Aperio ImageScope (Leica Biosystems Inc.). The section pairs were opened in the program side by side at a final magnification of 100–120×. Both images were moved simultaneously to ensure the same frame was seen in both pairs, and the lookup section was constantly adjusted to match the reference section using a 3 cm × 3 cm grid placed over the top the images. Glomeruli were identified in both sections. Glomeruli present in the *n*th section that were no longer present in the *n*th* *+* *2 section were marked as disappearing glomeruli, and the glomeruli present in the *n*th* *+* *2 section that were not present in the *n*th section were marked as appearing glomeruli, as demonstrated in Figure 1. This process was then repeated for each complete pair of sections and the sum of all disappearing and appearing glomeruli from the section pairs (*Q* ^−^) was used to calculate the total glomerular number per kidney using the following equation:

$$N_{\mathrm{glom}}=\frac{1}{\text{SSF}}\times \frac{1}{2(\;f(a))}\times Q^{-},$$

where *N*_glom_ is the total number of PNA‐positive glomeruli in the kidney, 1/SSF is the reciprocal of the section sampling fraction (the number of sections advanced between pairs), 1/2 *f*(*a*) is the inverse of the fraction of total section area used to count glomeruli, where 2 refers to the fact that disectors were used to count in both directions, *f*(*a*) refers to the fractional area, and *Q* ^*‐*^ is the total number of appearing and disappearing PNA‐positive structures between the reference and lookup sections for the kidney.

### Kidney volume

Kidney volume was estimated using the Cavalieri principle using the following formula:

$$V_{\mathrm{kid}}=\Sigma P\times a\left(p\right)\times t\times 1/f,$$

where *V*_kid_ is the kidney volume, Σ*P* is the total number of grid points (*p*) counted, *a(p)* is the area associated with each point, *t* is the section thickness, and *1/f* is the reciprocal of the section sampling fraction.

### Glomerular volume

Mean glomerular volume (*V*_*glom*_) was estimated as previously described (Cullen‐McEwen et al. 2003, 2011, 2012) using the following formula:

$$V_{\mathrm{glom}}=\left(\frac{P_{\mathrm{glom}}}{P_{\mathrm{kid}}}\right)/\left(\frac{N_{\mathrm{glom}}}{V_{\mathrm{kid}}}\right),$$

where *N*_glom_ is the total number of glomeruli per kidney, *P*_kid_ is the number of grid points on kidney tissue, *P*_glom_ is the number of grid points on glomerular tuft, and *V*_kid_ is the total kidney volume.

Total glomerular volume *(V*_glomtotal_ *)* was then calculated as *V*_glom_ × *N*_glom_.

### Glomerular morphology

Mesangial matrix expansion, the predominant glomerular histopathological change with age, was assessed both quantitatively and semiquantitatively as previously reported (Zhang et al. 2012, 2017). In brief, kidney sections that had been stained with PAS were scanned at 20x using an Aperio AT2 Scanner (Leica Biosystems Inc., Lincolnshire, IL), digitized, and analyzed in a masked fashion with a computer‐assisted image analysis program (Aperio ImageScope, Leica Biosystems Inc.). Fifty randomly selected glomeruli from each kidney section were assessed, with mesangial matrix quantified as the proportion of the glomerular tuft that was occupied by PAS‐positive material.

Mesangial expansion was assessed using a semiquantitative technique in a masked fashion, as whereby the degree of mesangial expansion in each glomerulus was quantified on PAS‐stained sections, grading them on a scale of 0–4: Grade 0, normal; Grade 1, area up to 25% (minimal); Grade 2, area 25–50% (moderate); Grade 3, area 50–75% (moderate to severe), and Grade 4, area 75–100% (severe). A mesangial expansion index (MEI) was then calculated using the formula:

$$\text{MEI}=\sum_{i=0}^{3}Fi(i),$$

where *Fi* is the percentage of glomeruli in the mouse with a given score (*i*).

### Statistical analysis

All data are shown as mean ± SEM unless otherwise specified. Between‐group differences were analyzed using a one‐way ANOVA with a post hoc Fisher's least significant difference test. Differences between repeated measures from a single animal were analyzed using a repeated measures ANOVA. All statistical analyses were performed using GraphPad Prism 6.0 for Mac OS X (GraphPad Software, San Diego, CA). A change was considered statistically significant if *P* < 0.05.

## Results

### Deacetylase is reduced in Sirt1^Y/Y^ mice

**Figure 2:**
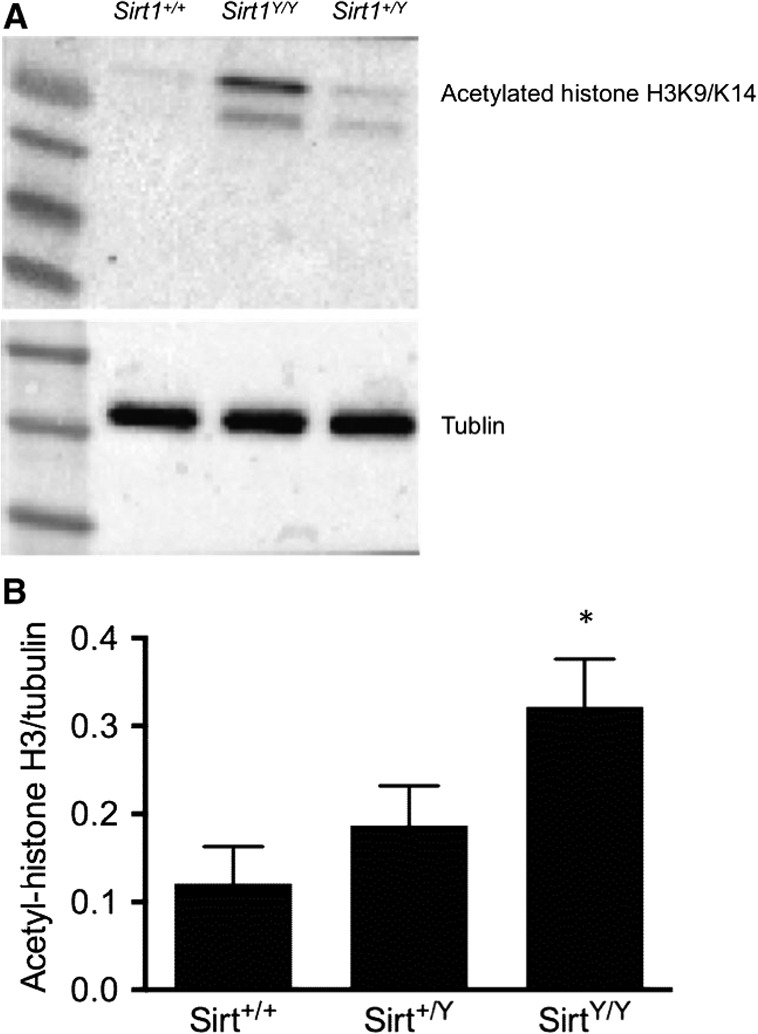
Acetylated histone H3K9/K14 protein expression in the kidneys of 14‐month‐old wild‐type (*Sirt1* ^*+/+*^), homozygous catalytically inactive (*Sirt1* ^*Y/Y*^) and heterozygous (*Sirt1* ^*+/Y*^) mice. Representative immunoblot of the kidneys from *Sirt1* ^*Y/Y*^ mice show increased histone H3K9/K14 acetylation (A) along with its quantitation relative to tubulin (B). *P < 0.05.

The acetylation status of histone H3 at lysine residues 9 and 14 was assessed to provide an index the enzymatic deacetylase activity of SIRT1. Notably, histone H3 when acetylated at K14 is also a marker of senescence (Xu et al. 2016). Consistent with the animals’ genotype, acetylation level of histone H3K9/K14 was increased in the kidneys of *Sirt1* ^*Y/Y*^ mice when compared with their wild‐type counterparts (Fig. 2).

### Older *Sirt1* ^*Y/Y*^ mice have smaller kidneys

When compared with their wild‐type counterparts at 14 months, SIRT1 catalytically inactive *(Sirt1* ^*Y/Y*^) mice weighed 20% less, while their kidneys weighed 39% less. These differences remained significant following adjustment for body weight (Table 1). While food intake was similar in wild‐type and *Sirt1* ^*Y/Y*^ mice, body temperature was significantly lower in the latter in association with a numerical trend toward lower activity (Table 1). No difference between wild‐type and heterozygous *Sirt1* ^*+/Y*^ was noted in any of the above.

**Table 1:** Animal characteristics at 14 months

	*Sirt1* ^*+/+*^	*Sirt1* ^*+/Y*^	*Sirt1* ^*Y/Y*^
Body weight (g)	43.6 ± 1.6	40.9 ± 2.2	34.9 ± 1.7*, †
Kidney weight (g)	0.33 ± 0.02	0.33 ± 0.02	0.20 ± 0.01*, †
Kidney: body weight ratio	0.76 ± 0.04	0.80 ± 0.03	0.58 ± 0.02*, †
Kidney volume (mm^3^)	239 ± 21	221 ± 12	152 ± 13*, †
Food intake (g/day)	5.2 ± 0.2	5.3 ± 0.3	4.8 ± 0.2
Body temperature (°C)	33.8 ± 0.2	33.8 ± 0.4	33.3 ± 0.2*
Activity counts (counts/day)	7130 ± 2204	6390 ± 1715	5190 ± 1741
eSNGFR (nL/min)	9.2 ± 0.9	10.1 ± 0.9	7.1 ± 0.6
Urinary albumin excretion rate (*μ*g/day)	83.8 ± 38.7	90.2 ± 46.0	84.8 ± 31.0

All values are expressed as mean ± SEM.

* *P* < 0.05 versus *Sirt1* ^*+/+*^.

† *P* < 0.05 versus *Sirt1* ^*+/Y*^.

### Older Sirt1^Y/Y^ mice have fewer glomeruli and lower GFR

**Figure 3:**
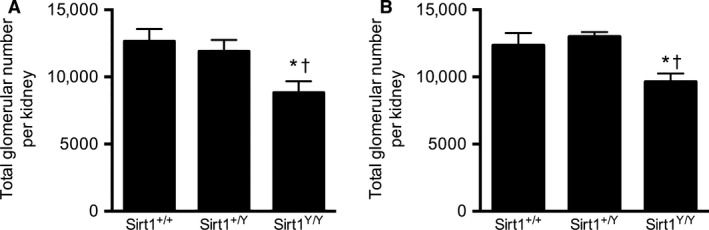
Total glomerular number in 14‐month‐old (A) and 4‐week‐old (B) male mice. *P < 0.05 versus *Sirt1* ^*+/+*^, ^†^ *P* < 0.05 versus *Sirt1* ^*+/Y*^.

**Figure 4:**
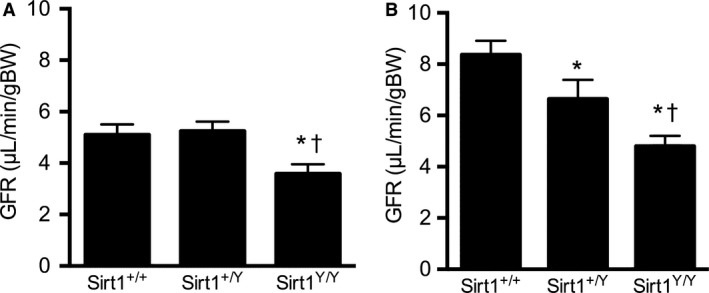
Glomerular filtration rate (GFR) in 14‐month‐old (A) and 4‐week‐old (B) male mice. *P < 0.05 versus *Sirt1* ^*+/+*^, ^†^ *P* < 0.05 versus *Sirt1* ^*+/Y*^.

To determine if lack of SIRT1 activity plays a role in determining total glomerular number, glomeruli were counted using the disector/fractionator combination. Fourteen‐month *Sirt1* ^*Y/Y*^ mice had 30% fewer glomeruli compared with their wild‐type and heterozygous *Sirt1* ^*+/Y*^ counterparts (Fig. 3). Consistent with the lower glomerular number, GFR, as assessed by FITC‐inulin clearance, was also reduced by 29% in the *SirtI* ^*Y/Y*^ mice with heterozygous *Sirt1* ^*+/Y*^ showing a GFR that was similar to wild‐type mice (Fig. 4).

Estimated single nephron GFR (eSNGFR) was calculated by dividing GFR by twice the number of glomeruli in a single kidney. With this equation, the estimated mean eSNGFRs were similar among groups (Table 1).

Urinary albumin excretion rate, assessed over a 24‐hour period, was also similar in the three groups (Table 1).

### SIRT1 activity does not affect glomerular structure

**Figure 5:**
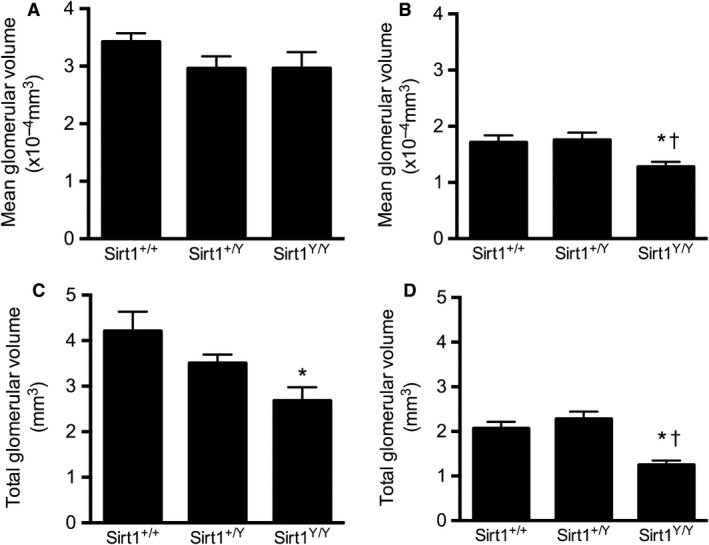
Mean glomerular volume (×10^−4 ^mm^3^) in 14‐month‐old (A) and 4‐week‐old (B) male mice and total glomerular volume (mm^3^) in 14‐month‐old (C) and 4‐week‐old (D) male mice. *P < 0.05 versus *Sirt1* ^*+/+*^, ^†^ *P* < 0.05 versus *Sirt1* ^*+/Y*^.

**Figure 6:**
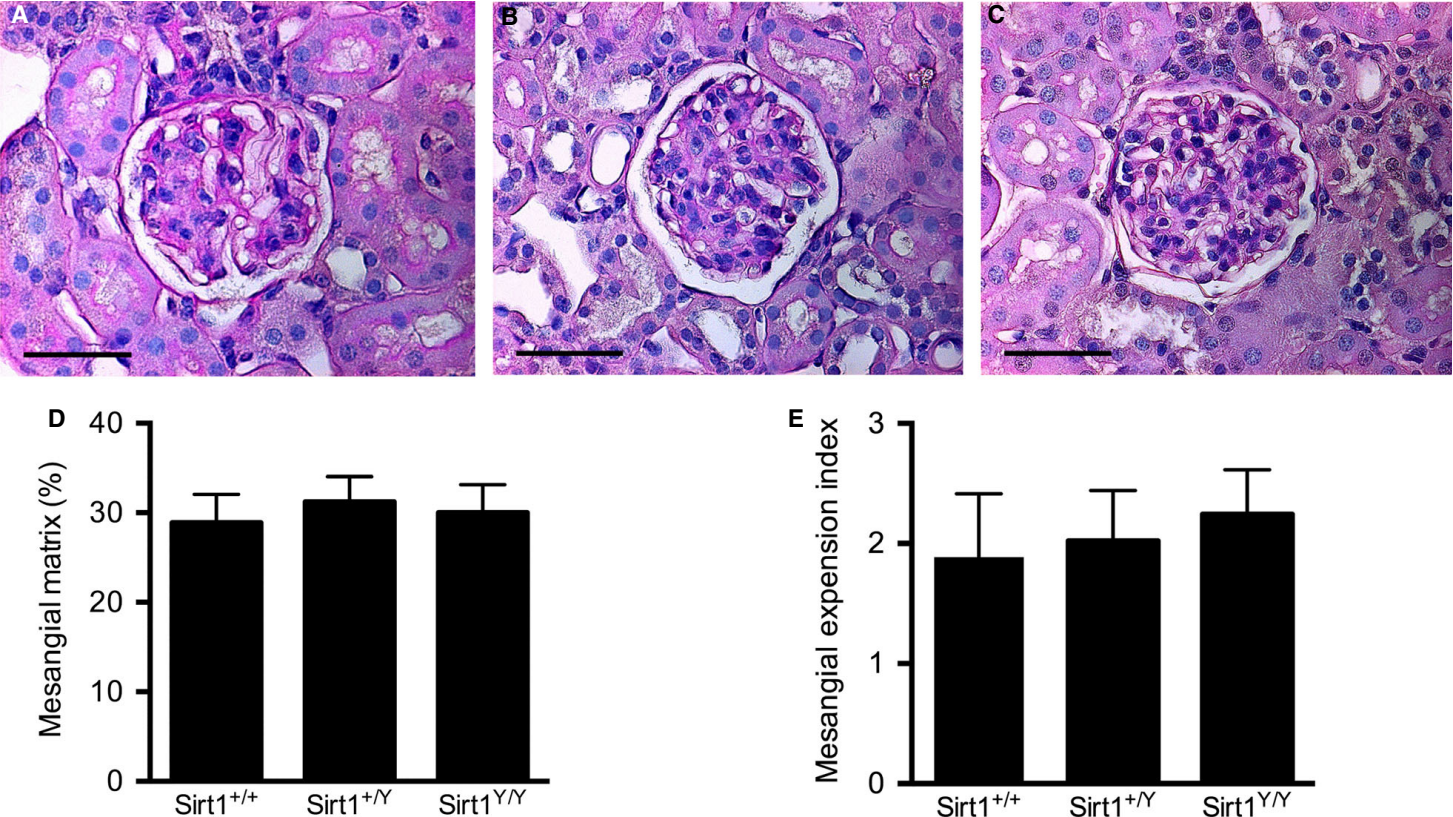
Mesangial matrix expansion in 14‐month‐old male mice. Kidney sections were stained with PAS to assess the proportion of mesangial matrix within the glomeruli. Representative images of glomeruli from (A) wild‐type mice, (B) heterozygous *Sirt1* ^*+/Y*^ mice, and (C) catalytically inactive *Sirt1* ^*Y/Y*^ mice. Percentage area of glomerular tuft occupied by mesangial matrix (D) and mesangial expansion index (MEI, E). Scale bar = 50 *μ*m.

In consideration of the lower GFR in the catalytically inactive *Sirt1* ^*Y/Y*^ mice, glomerular structure was assessed in PAS‐stained sections. Glomeruli did not show evidence of hypertrophy with similar estimated mean glomerular volumes in wild‐type and catalytically inactive *Sirt1* ^*Y/Y*^ mice (Fig. 5). The total glomerular volume for the catalytically inactive Sirt1^Y/Y^ mice was significantly lower than the wild‐type mice, consistent with their lower glomerular number (Fig. 5). Likewise, we found no evidence of increased sclerosis or mesangial expansion between groups as assessed by the proportion of the glomerular tuft occupied by PAS‐stained material by either quantitative or semiquantitative techniques (Fig. 6).

### Young and aged *Sirt1* ^*Y/Y*^ mice display common attributes

To determine whether the lower GFR and glomerular number in *Sirt1* ^*Y/Y*^ mice were a consequence of accelerated aging with associated glomerular loss or the result of reduced glomerular endowment, young, 4‐week‐old mice were studied. Like older mice, kidney: body weight ratios were also substantially lower in young *Sirt1* ^*Y/Y*^ mice (Table 2). Glomerular number and GFR (Figs. 3, 4) were similarly also lower in 4‐week‐old catalytically inactive *Sirt1* ^*Y/Y*^ mice than in their wild‐type counterparts. Mean glomerular volume and total glomerular volume were also lower in the catalytically inactive Sirt1^Y/Y^ mice (Fig. 5).

**Table 2:** Animal characteristics at 4 weeks

	*Sirt1* ^*+/+*^	*Sirt1* ^*+/Y*^	*Sirt1* ^*Y/Y*^
Body weight (g)	26.5 ± 0.8	25.5 ± 0.5	19.3 ± 0.9*, †
Kidney weight (g)	0.27 ± 0.02	0.23 ± 0.01	0.10 ± 0.004*, †
Kidney: body weight ratio	0.92 ± 0.04	0.87 ± 0.04	0.53 ± 0.01*, †
Kidney volume (mm^3^)	152 ± 10	155 ± 10	99 ± 11*, †

All values are expressed as mean ± SEM.

* *P* < 0.05 versus *Sirt1* ^*+/+*^.

† *P* < 0.05 versus *Sirt1* ^*+/Y*^.

**Figure 7:**
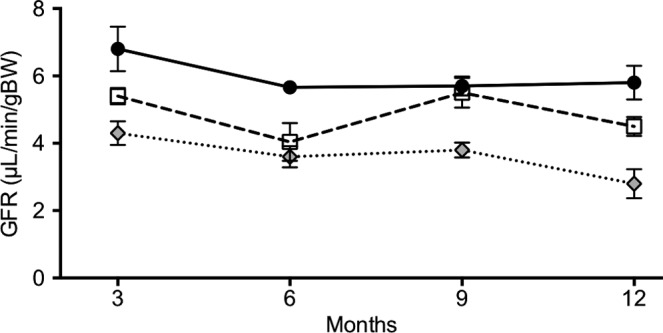
Longitudinal changes in GFR in *Sirt1* ^*+/+*^(black circle), *Sirt1* ^*+/Y*^ (white square), and *Sirt1* ^*Y/Y*^ (gray diamond) mice.

In the subgroup of animals in which GFR had been measured longitudinally at 3‐monthly intervals for 1 year, a lower baseline GFR was noted among catalytically inactive *Sirt1* ^*Y/Y*^ mice. The rate of GFR decline, however, did not differ between groups at −1.87 ± 0.81, −1.09 ± 0.11, and −1.86 ± 0.75 (mean ± SEM) *μ*l/min/gBW/year in *Sirt1* ^*+/+*^, *Sirt1* ^*+/Y*^, and *Sirt1* ^*Y/Y*^ mice, respectively (Fig. 7).

## Discussion

While the majority of studies investigating SIRT1 in mammalian biology indicate a key role in the aging process, this study suggests that its predominant effect was, rather, an effect on glomerular endowment. While glomerular hypertrophy and elevated single nephron GFR traditionally accompany reduced glomerular number, this was not seen in catalytically inactive SIRT1 mice, suggesting that these phenomena may be SIRT1 dependent.

Calorie restriction is one of the few interventions that has been shown to increase life span, doing so in a variety of organisms that range from unicellular eukaryotes to nonhuman primates (Fontana and Partridge 2015). As a corollary, energy excess, manifest as increased body weight is, in general, associated with reduced life expectancy in rodents (Sriram et al. 2002) and humans (Calle et al. 1999). Though the study of the relationship between calorie intake and aging is in its infancy, several lines of investigation indicate that the lysine deacetylase, SIRT1, may play a pivotal role (Bordone and Guarente 2005). In the kidney, the age‐associated reduction in SIRT1 that can be attenuated by calorie restriction (Kume et al. 2010) also reduces the structural and functional manifestations of kidney aging in rodent models of kidney aging (Jiang et al. 2005). Similarly, 8 weeks of calorie restriction was found to markedly reduce glomerular hypertrophy, fibrosis, and proteinuria in aged, 25‐month‐old rats in association with elevated levels of SIRT1 (Ning et al. 2013). Furthermore, other studies using a pharmacological approach to enhance SIRT1 activity have also shown attenuation of experimental kidney injury (Zhang et al. 2017). Accordingly, we hypothesized that SIRT1 inactivation, as examined in this study, would accelerate the structural and functional manifestations of kidney aging. The finding that this was not the case illustrates the differences between genetic defects, present at the time of conception, and the use of diet and pharmacological approaches in adult animals. By studying animals with a genetically determined, catalytically inactive SIRT1, this study is able to directly examine the effects of the enzyme without the confounding effects induced by dietary manipulation or the uncertainties surrounding the sirtuin‐activating compounds (Milne et al. 2007; Pacholec et al. 2010). Here, we show that while SIRT1 inactivity led to diminished nephron endowment, it was followed by neither glomerular enlargement, nor elevated eSNGFR nor an accelerated rate of GFR decline with age.

In humans, nephron number varies inversely with glomerular volume where in the setting of fewer nephrons, glomeruli undergo hypertrophy with consequent single nephron hyperfiltration. As proposed by Brenner and colleagues almost 30 years ago (Brenner et al. 1988), these initially compensatory changes may ultimately be maladaptive whereby single nephron hyperfiltration leads to glomerular damage and ultimately faster rates of GFR decline (Lemley 2003; Denic et al. 2017b). As such, reduction in glomerular number, regardless of whether congenital or acquired, was proposed to lead to an inexorable decline in kidney function with time (Hostetter et al. 1982). This phenomenon is well illustrated in the remnant kidney model in the rat. While the hypertrophy and accompanying single nephron hyperfiltration that develop in this model afford initial compensation for the loss of 5/6 of its renal mass, within a matter of weeks, progressive glomerulosclerosis, declining GFR and uremic death supervene (Wu et al. 1997). In contrast, despite a congenital reduction in nephron number, the glomeruli of *Sirt1* ^*Y/Y*^ mice failed to undergo compensatory hypertrophy or increase in eSNGFR that may explain why the rate of GFR decline and the development of structural pathology were similar in wild‐type and catalytically inactive SIRT1 animals. Furthermore, the absence of glomerular hypertrophy or elevated eSNGFR in response to reduced nephron number raise the possibility that SIRT1 may directly or indirectly contribute to this initially compensatory but ultimately detrimental response.

This study has limitations. First and foremost, it is a study of experimental animals not humans. Moreover, background mouse strain is an important determinant of the response to kidney injury and while the common laboratory mouse strain, C57BL/6, is notoriously resistant to developing kidney disease, CD1 mice, as used in this study, are far more susceptible, developing phenotypes more reminiscent of human disease (Sugimoto et al. 2007; Leelahavanichkul et al. 2010). With glomerular endowment as a key parameter measured in this study, the accurate ascertainment of glomerular number was pivotal. As such, we used the disector/fractionator method that, while tedious and time consuming, provides unbiased results to have become the gold standard for glomerular counting supplanting older, biased methods (Cullen‐McEwen et al. 2011, 2012). This study explored global SIRT1 inactivity contrasting the very recent report by Chuang and colleagues who explored the effects of podocyte‐specific SIRT1 shRNA‐based knockdown in aging mice, reporting dramatic increases in glomerulosclerosis, albuminuria, and BUN (Chuang et al. 2017). Notably, however, in the latter, podocyte‐specific knockdown study, mice that expressed control luciferase shRNA also developed marked glomerulosclerosis and increased BUN suggesting a prominent, nonspecific effect of the shRNA. And while the current study did not assess the role of SIRT1 in nephrogenesis, recent publications attest to the role of SIRT1 substrates, p53 and histone H3K9/K14 in nephron development (El‐Dahr et al. 2014; El‐Dahr and Saifudeen 2018; Liu et al. 2018). Finally, the effects of superimposing kidney stressors such as diabetes or hypertension were not assessed.

In summary, this study supports a role for SIRT1 in the determination of nephron endowment and in the compensatory glomerular enlargement and single nephron hyperfiltration that would otherwise accompany reduced glomerular number.

## Conflict of Interest

None declared.
